# Fabrication and evaluation of a novel patient-specific 3D-printed simulation model for oral surgical training

**DOI:** 10.1186/s12909-025-08402-1

**Published:** 2025-12-09

**Authors:** Leila Gholami, Edward Putnins, HsingChi von Bergmann, Arvin Bagheri, Rana Tarzemany

**Affiliations:** 1 Department of Oral Biological and Medical Sciences, Faculty of Dentistry, The University of British Columbia, Vancouver, BC Canada; 2 Department of Oral Health Sciences, Faculty of Dentistry, The University of British Columbia, Vancouver, BC Canada; 3 Nobel BioCare Oral Health Centre, Faculty of Dentistry, The University of British Columbia, Vancouver, BC Canada

**Keywords:** Dental education, Simulation, Model fabrication, 3D printing, Oral surgery, Flap reflection

## Abstract

**Background:**

Use of patient-specific models as a surgical planning and training tool can support novice practitioners’ surgical skill development. This study aimed to introduce a novel workflow for fabricating 3D-printed, patient-specific simulation models and evaluate their accuracy and transferability for use in periodontal and oral surgery training.

**Methods:**

Patient-specific anatomical models of the maxilla were fabricated using the CBCT and intraoral scan data. The proposed workflow outlines a novel process for creating a patient-specific model that accurately replicates both the hard and soft tissues of the patient. The accuracy of the printed models was evaluated by scanning five models and comparing them to the patient’s intraoral scan using cloud-to-cloud distance analysis. Then, in an exploratory study design, a simulated gingival flap surgery exercise was completed by 18 periodontists and 50 students. The face and content validity of the model were assessed using an 8-item online questionnaire with a VAS of 0-100 and a free comment question. The data were analyzed using descriptive statistics, Mann–Whitney U tests and independent t-test.

**Results:**

The printed model demonstrated high dimensional accuracy. The overall VAS score of the model was significantly higher for students than for periodontists (83.7 ± 9.7 vs. 72.1 ± 15.8, *p* < 0.006). The face and content validity scores reported by students were also higher (*P* < 0.01), with mean differences of 8.86% and 12.62%, respectively. Periodontists rated the models lower for soft-tissue tactile feedback, particularly during incision.

**Conclusions:**

The proposed 3D-printed simulation workflow produced an accurate and educationally valuable model with the potential to enhance surgical training. Experienced surgeons suggested that refining the soft-tissue realism could further improve its overall educational value.

## Background

Use of three-dimensional (3D) simulation models has become more common in contemporary healthcare education in recent years due in part to advances in 3D printing technologies. For example, to better prepare novice surgeons, medical simulation models are used, where trainees practice procedures and complete complex tasks before transitioning to patient-based surgeries [1–3]. Oral surgical training for dental students has traditionally relied on various training models, including typodonts and cadaveric animal models. While these models have been invaluable for hands-on practice, they have limitations, including differences from human anatomy, ethical considerations, and issues related to storage and infection control. Recently, 3D imaging and printing technologies have introduced a new dimension to simulation-based education: the creation of patient-specific anatomical models. With the integration of cone-beam computed tomography (CBCT) and intraoral scans data, these models can replicate individual patient hard and soft tissue anatomy. This provides a more realistic learning experience, allowing students to practice and refine their skills in a controlled environment that mimics a real-life scenario [4–6].

Their application has gained increasing attention for both preoperative planning and hands-on education, offering tangible, anatomically accurate structures for students and clinicians to examine and operate on. This enhances the understanding of anatomical structures, procedural steps, and planning for potential complications, particularly in complex or uncommon clinical scenarios [7]. Moreover, 3D-printed anatomical replicas have shown promise in improving patient education, assisting with shared decision-making, and enabling the customization of surgical tools and implantable materials [8–10].

Numerous efforts have been made to replicate precise patient-specific anatomical models, and many authors have developed and presented their versions of simulation models using a variety of printers and materials [11–18]. These models were first used in cranio-maxillofacial surgery, but their use has since expanded to other areas of oral surgery. Accurate replication of precise oral soft tissues with appropriate tactile properties has been challenging, and various techniques have been explored to improve soft tissue simulation. However, many of them are not 3D printed methods designed to replicate the patient’s exact soft tissues [11, 16, 19, 20]. Moreover the use of intraoral scanning data for accurate soft-tissue replication has received limited attention in the available literature.

While 3D printing has been widely adopted for surgical planning, its application in dental education, particularly in periodontology and intraoral surgery, remains underexplored.

Dental students in their final clinical years often face limited opportunities to perform complex surgical procedures. Fabrication of patient-specific models that support safe, realistic simulation-based training can offer a valuable platform for developing surgical skills and bridging the gap between theoretical learning and clinical experience in intraoral surgery for undergraduate and graduate level dental students.

In this study, we present a novel workflow for in-house fabrication of a craniofacial pedagogical simulation model for intraoral surgery using readily available dental 3D printers and materials. By integrating intraoral scans with CBCT data obtained from the patient, precise replicates of both the hard and soft tissues of the patient can be created [9].

Additionally, we aimed to assess the accuracy of this method in replicating patients’ hard and soft tissues and to explore its face and content validity through evaluations by students and expert periodontists. Assessing these models from both learner and expert perspectives provides valuable insight into their educational utility.

## Methods

Authorization to utilize anonymized patient imaging data from the university database and to undertake all aspects of this project was granted by the University of British Columbia Research Ethics Board (H24-02135). The research was conducted in accordance with the Declaration of Helsinki and national/institutional standards. Written informed consent was obtained from all participants. The questionnaire responses and evaluation data contained no personal identifiers.

This was an exploratory study conducted among final-year dental students and periodontist faculty instructors within our faculty of dentistry to evaluate the feasibility and educational value of 3D-printed models designed for intraoral surgery training.

### Model design and fabrication

CBCT data from a patient requiring bone grafting at site #24, obtained with a CBCT imaging system (J. Morita Corp., Kyoto, Japan), were used. Intraoral scans of the same patient were also obtained to record the patient’s gingival tissue using the TRIOS 3 scanner (3Shape A/S, Copenhagen, Denmark). DICOM files from the CBCT scan were imported into the dental implant planning software, CoDiagnostiX (Dental Wings GmbH, Chemnitz, Germany), for 3D volumetric reconstruction and were exported in standard tessellation language (STL) format. The STL files were then processed in Meshmixer software (Autodesk, USA) for segmentation and refinement. Non-relevant anatomical structures were removed, and a minimal smoothing algorithm was applied to preserve essential anatomical features while reducing digital noise.

The digital file was used as the “Bone” model to represent the hard tissues of the jaw, including both bone and teeth, for 3D printing. Two mounting pins were incorporated into the model to facilitate its assembly on a dedicated base compatible with a mannequin head. The STL file obtained from the intraoral scanner was subsequently merged with the “Bone” model to generate a combined “Bone + Soft Tissue” (B + S) model, which served as the foundation for the design of a novel injection mould. Using Meshmixer, a mould block was constructed around the B + S model. This block was divided horizontally into two sections (top and bottom) using the “Plane Cut” command, after which the B + S model was subtracted from each section via the “Boolean Difference” operation. To optimize both the injection process and the removal of moulded parts, the top section was further segmented into smaller parts using the same “Plane Cut” tool. Injection gates were incorporated as four mm-diameter cylindrical channels, strategically positioned to ensure complete exposure of all target surfaces to the soft-tissue material. These gates were created using the “Boolean Difference” command.

**Fig. 1:**
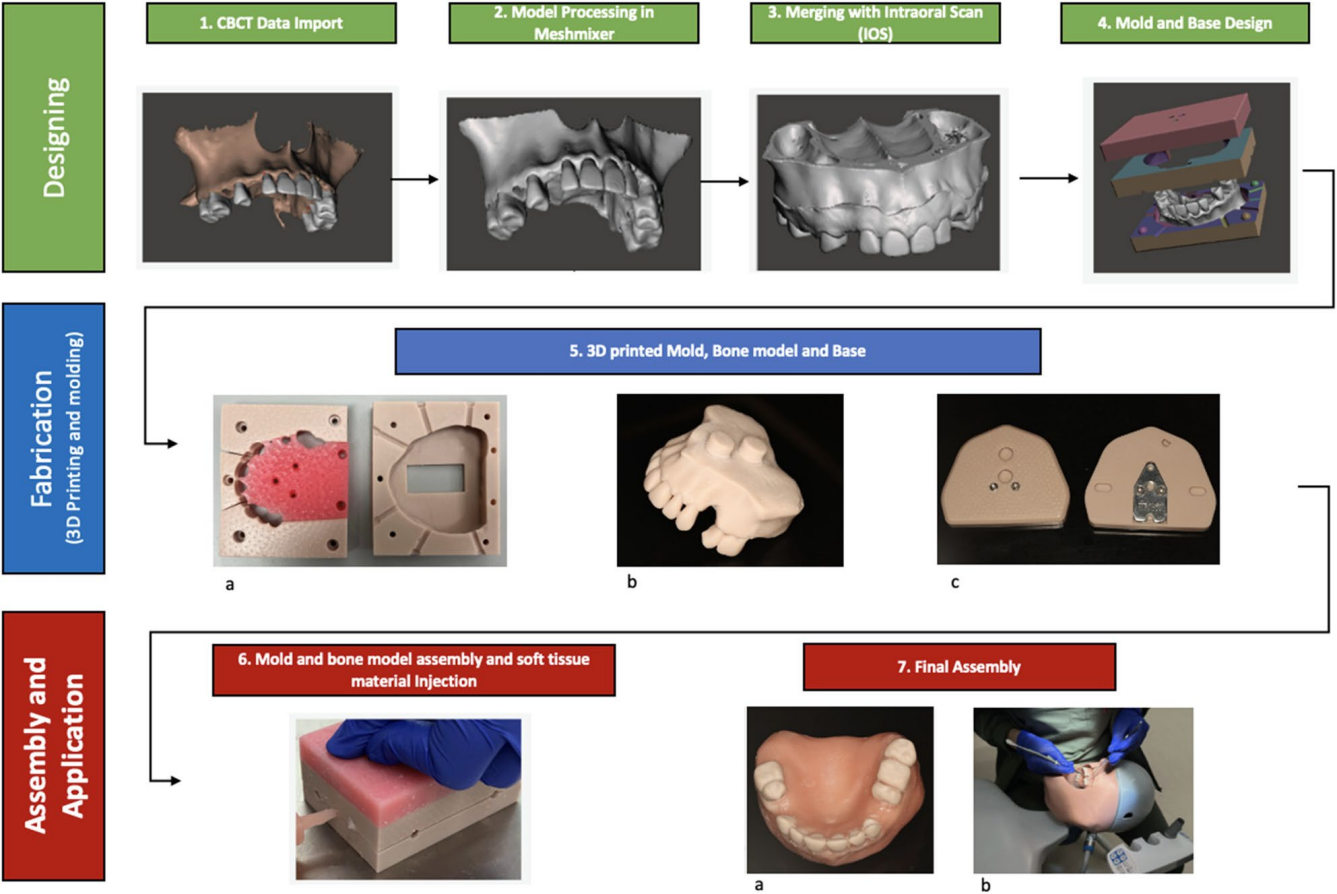
3D-printed simulation model fabrication workflow: 1) CBCT data were imported into CoDiagnostix software to generate a 3D model of the patient’s maxillary bone. 2) The 3D bone model was exported to Meshmixer for artifact removal, trimming of unnecessary areas, and conversion into a solid structure. 3) The solid bone model was merged with the patient’s intraoral scan, resulting in a composite model representing bone covered with soft tissue (Bone and soft tissue model). 4) A mould was designed around the composite model, including injection gates and fastening holes. A base was also designed to allow mounting the bone model into a mannequin head. 5) 3D printing of the designed mould (**a**), bone model (**b**) and mounting base (**c**). 6) The bone model was placed inside the mould, and the components were assembled to allow soft tissue material injection through the designed gates. 7) The cured model was removed from the mould, cleaned, and any excess material was trimmed (**a**). The completed model was mounted on the base and placed inside the mannequin head for further assessment and use in surgical training or evaluation (**b**)

Additionally, 3 mm-diameter screw holes were incorporated into the mould design to allow secure fastening and separation of the mould halves (Fig. 1). A clearance space was included in the bottom section to accommodate the mounting pins of the “Bone” model without interference. Finally, a base modelled after the standard Frasaco typodont system featuring two pin holes compatible with the designed mounting pins was developed. (Fig. 1).

A SprintRay Pro95 3D printer (SprintRay Inc., Los Angeles, CA, USA) was used to print the hard-tissue model, the soft-tissue mould, and a mounting base to attach the model to a Frasaco dental simulation manikin head. The 3D hard-tissue model and mould were printed with SprintRay Die and Model 2 resin. In contrast, some parts of the mould were printed with SprintRay Gingiva Mask resin for greater flexibility during disassembly. To identify the best material to use to simulate the soft tissue layer, we evaluated three potential materials for soft tissue simulation: (i) Coe-Soft self-curing reline material (GC America Inc., Alsip, IL, USA), selected for its favourable stretchability; (ii) Gingiva Mask (SprintRay, USA), the soft resin provided by the printer manufacturer; and (iii) GI-Mask Silicone (Coltene Whaledent AG, Altstätten, Switzerland), a commonly used material for fabricating oral soft tissue models. Two experienced clinicians (each with more than 10 years of clinical practice) independently assessed the materials across four criteria, stretchability, appearance, incision tactile sensation, and suturability, using a 5-point Likert scale. The material achieving the highest mean rating across these categories was selected for subsequent model fabrication.

This soft-tissue material was injected into the printed mould to create the soft tissues overlaying the model’s bone structure. Once cured, the model was removed from the mould, cleaned, and checked for defects. Any excess material was trimmed (Fig. 1).

### Model fabrication cost

A cost analysis based on material usage and unit prices was conducted to estimate the cost of producing a single patient-specific full-arch model incorporating both hard and soft-tissue (gingiva) materials.

### Quantitative evaluation of model accuracy

The accuracy of the fabricated model was quantitatively evaluated by superimposing a scan taken from the final printed model onto the patient’s intraoral scan. Five identical models were printed using the described workflow. The discrepancies were assessed for each model using a heat map generated through cloud-to-cloud distance analysis in CloudCompare, an open-source software (Open-source 3D point-cloud analysis software; version 2.x, EDF R&D, France). The software calculated and reported the root mean square (RMS) of the discrepancies, which was considered as the measurement of print accuracy.

### Qualitative evaluations of the model

Using a questionnaire-based evaluation, we assessed the Face validity (authenticity), referring to the model’s visual realism, and Content validity (transferability), reflecting its perceived usefulness for skill development.

Eighteen periodontists (Faculty clinical instructors at the division of Periodontics) and fifty final-year undergraduate dental students were recruited voluntarily to participate in the study. In the expert group, seven participants had 5–10 years of clinical experience, while the remaining had more than 10 years of surgical and teaching experience in periodontics. The dental students had all participated in a clinical oral surgery course and had experience working with real human oral tissues.

Each participant was assigned an individual simulation model and surgical instrument set. Participants were instructed to perform a flap procedure, including incision, elevate the flap to visualize the bony defect at the edentulous site of #24 and suture the flap. The investigator was not present during the procedure to avoid bias but remained available to answer procedural or technical questions. Immediately following the exercise, participants completed an online evaluation questionnaire comprising eight questions designed to assess the model’s authenticity and transferability.

Participants recorded their responses on a continuous 0–100 Visual Analogue Scale (VAS) via the Qualtrics online survey platform. A sliding bar allowed participants to indicate their level of agreement, where zero represented “strongly disagree” and 100 represented “strongly agree.” The questionnaire concluded with a single open-ended question inviting participants to provide qualitative feedback or suggestions for improving the 3D-printed model (Table 1).

**Table 1:**
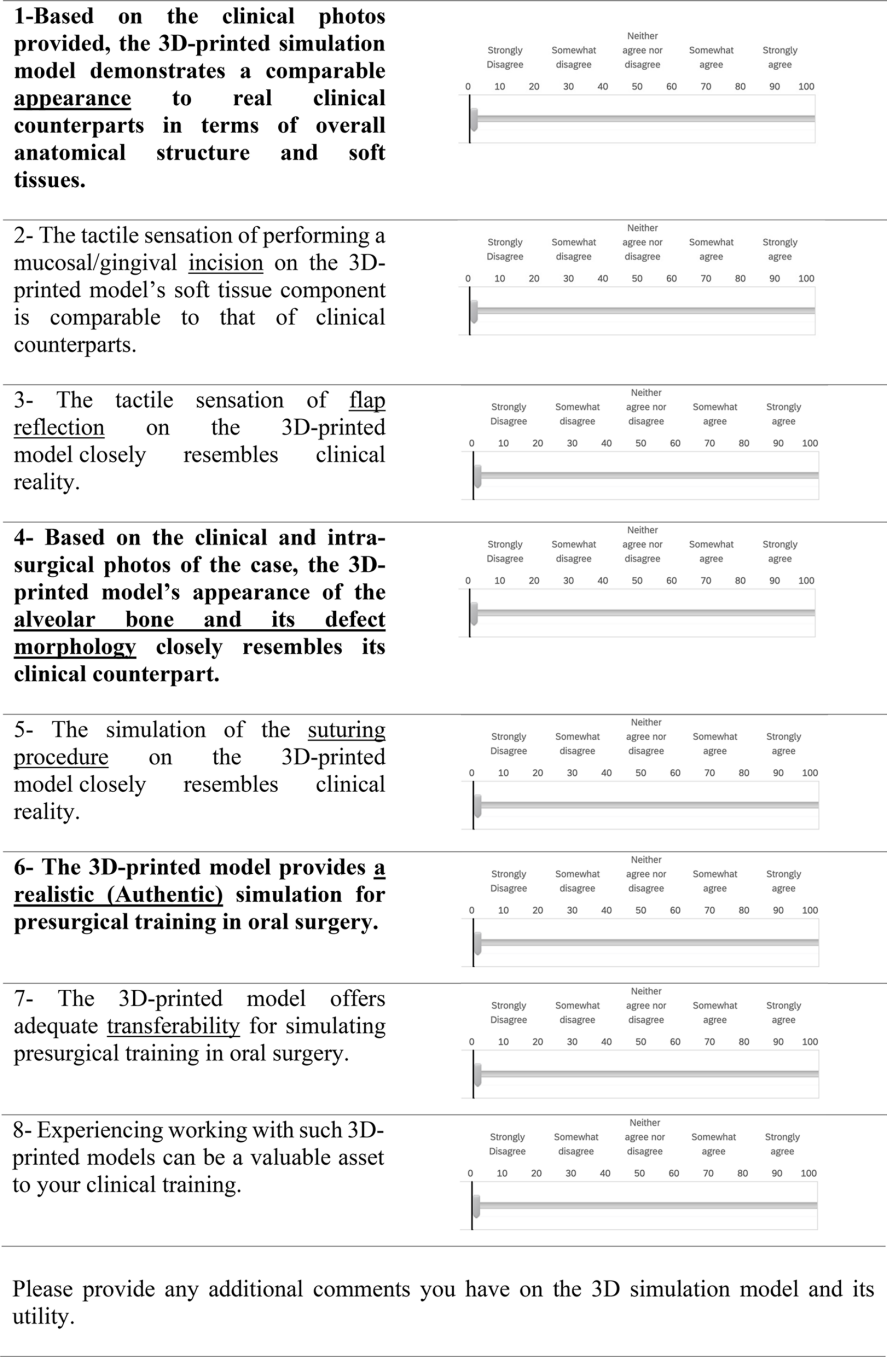
Model evaluation questionnaire

**Fig. 2:**
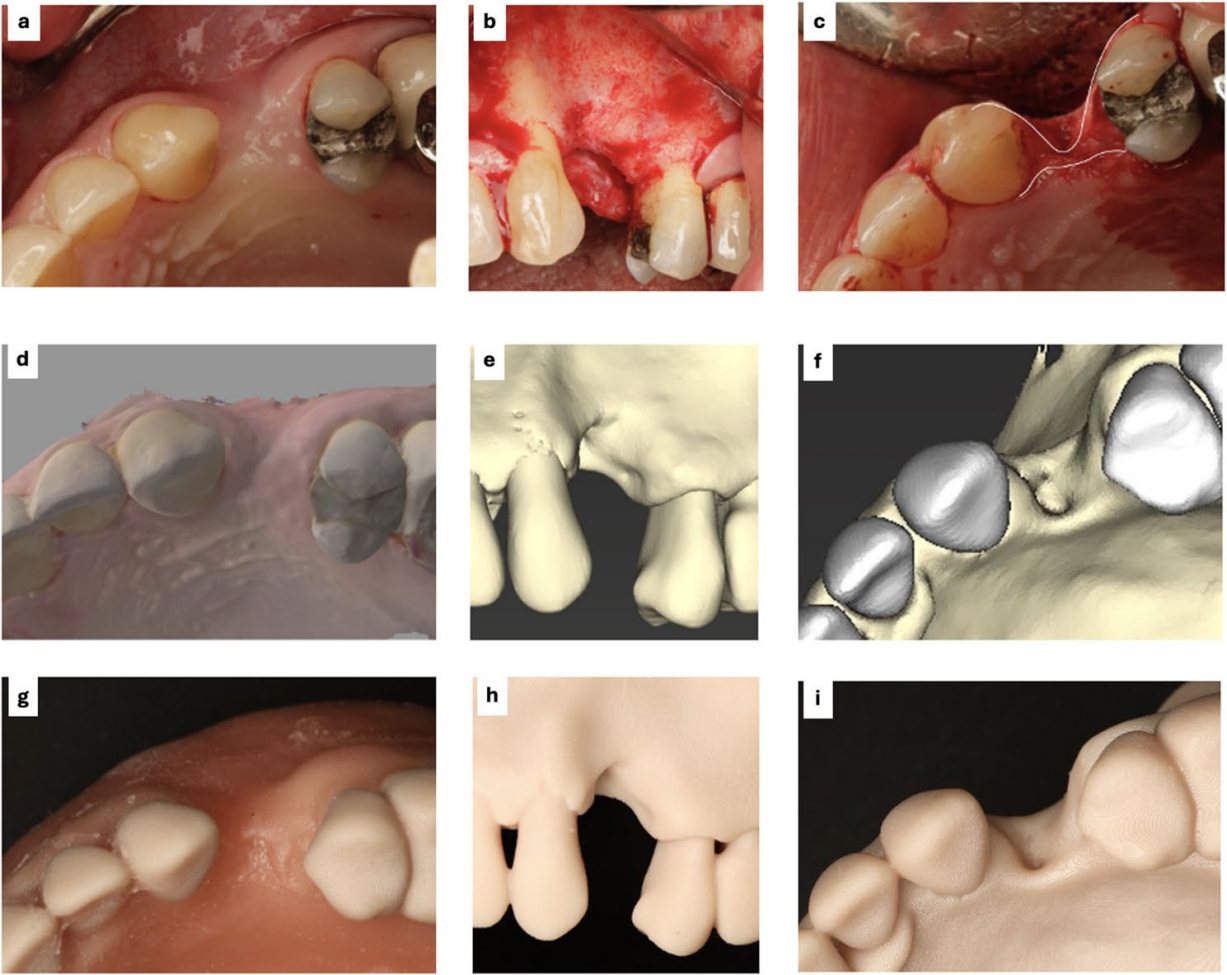
(**a**) Patient’s intraoral occlusal view photograph of the #24 defect site, **b** Buccal view of the site alveolar bone after flap elevation during surgery, **c** Occlusal view of the alveolar bone during surgery, **d** Intraoral scan showing the occlusal view of the #24 defect site, **e** and (**f**) Buccal and occlusal views of the defect site on CBCT images, respectively, **g** Final 3D-printed model showing the occlusal view of the #24 defect site, **h** and (**i**) Buccal and occlusal views of the 3D-printed bone model, respectively. Patient intraoral photos are courtesy of Dr. Sepehr Torabi

Intraoral, intra-surgical, and CBCT photos of the alveolar ridge defect at #24 were provided to participants to compare the 3D-printed model’s soft- and hard-tissue replicas with the patient’s photos (Fig. 2).

### Statistical analysis

This study was designed as an exploratory design to assess the feasibility and perceived educational value of 3D-printed models for oral surgery simulation training. All volunteered faculty instructors and final-year dental students were included using convenience sampling.

Descriptive statistics were performed for VAS responses. Mean scores and standard deviations and mean differences and 95% confidence intervals were reported for both student and periodontist groups.

Normality was assessed using the D’Agostino–Pearson test.Group comparisons were conducted using independent-samples t-tests for Q1–Q3, which met the normality assumption, and Mann–Whitney U and Welch t-test for Q4–Q8, where data were not normally distributed and account for unequal variances. Significance was assessed using a Bonferroni-adjusted threshold of *p* < 0.00625.

Subsequently, questions were grouped by thematic similarity into two domains: (1) model face validity (Q1, Q4, Q6) and (2) content validity (Q2, Q3, Q5, Q7, Q8). Composite scores were calculated by averaging responses within each domain. Independent-samples t-tests were used to compare the mean scores between student and periodontist participants. For face-validity and content-validity items, Bonferroni-adjusted threshold of < 0.01 was considered as significant.

All analyses were conducted using SPSS V.31(IBM Corp., USA) and GraphPad Prism V.10.5.0 for macOS (GraphPad Software, USA).

## Results

### Model fabrication

The workflow produced a realistic soft and hard tissue model (Fig. 1).

The sum of four ratings by each evaluator was averaged to serve as a voting method to choose the material. Based on this score, shown in Table 2, Coe-Soft self-curing reline material (GC America, USA) was chosen as it had the most suitable characteristics and achieved the highest mean score.

**Table 2:**
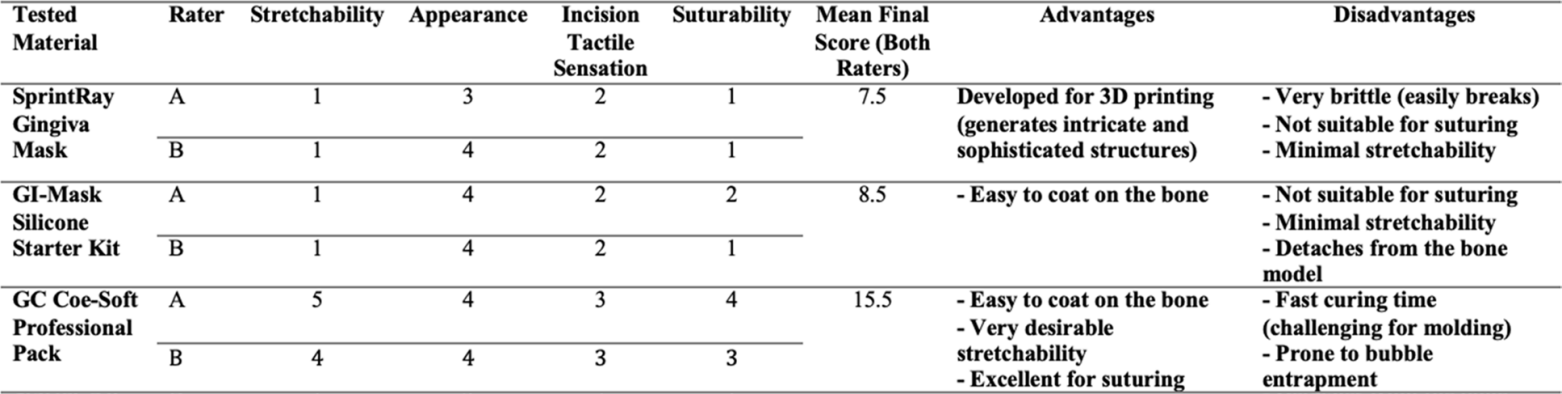
Evaluation of gingiva simulation materials by two independent raters. Each material was rated on four qualitative parameters (1–5 scale). The mean final score represents the average of both raters’ total scores. Advantages and disadvantages observed during evaluation are summarized

Coe-Soft offered excellent stretchability, a favourable tactile feel for incisions, and acceptable suturing performance, despite a minor drawback of fast-setting time. SprintRay Gingiva Mask, while optimized for 3D printing and capable of producing detailed structures, demonstrated minimal stretchability and poor suturing characteristics, resulting in a low final score. GI-Mask Silicone showed good stretchability and visual properties, however, it lacked good suturing capability. Scores between raters differed by no more than 1 point across all materials, indicating high agreement (Table 2).

The cost analysis estimated the material expense for fabricating a single patient-specific model using the proposed workflow to be approximately $60 USD for one full arch model.

### Quantitative evaluation of the 3D-printed model accuracy

**Fig. 3:**
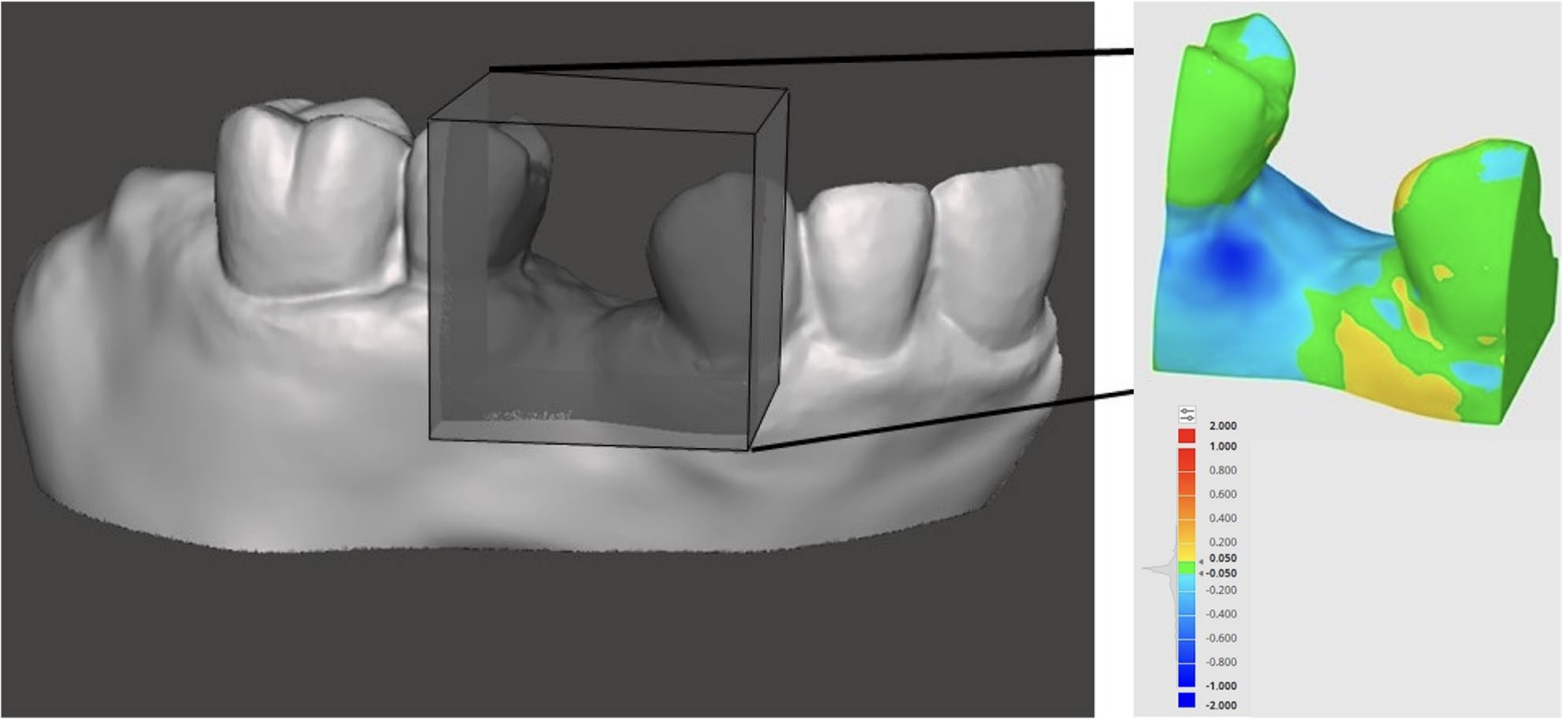
Cloud-to-cloud distance analysis was conducted in CloudCompare by superimposing the final printed model scan onto the original patient intraoral scan. The heat map illustrates surface deviations in millimetres, where blue indicates the least discrepancies and red indicates the greatest discrepancies between the model and the patient’s intraoral scan. Color scale representing surface deviation values ranging from − 2.000 mm (dark blue) to + 2.000 mm (red), where green indicates no deviation, 0 mm

Hard tissue structures were derived from CBCT data and 3D-printed using a printer with 100-micron precision. Anatomical accuracy of the printed models was evaluated by comparing them to the patient’s original scan data using cloud-to-cloud distance analysis in CloudCompare. The accuracy assessment of the five printed models, analyzed using CloudCompare, demonstrated high geometric fidelity relative to the original digital reference model. Across all samples, the average deviation ranged from − 0.05 mm to − 0.03 mm, while the root mean square error (RMS) was between 0.11 mm and 0.15 mm for all printed models, and the tolerance was less than 75% in all samples.These results showed that the fabrication method achieved an accuracy better than 200 microns, confirming its reliability for creating precise patient-specific training models. The models demonstrated adequate alignment, with blue indicating the least discrepancies and red indicating the greatest. (Fig. 3).

### Qualitative evaluations of the model

The students were all in their last year of dental education and had treated patients in the oral surgery course. Both periodontist and student groups agreed that the 3D-printed model demonstrated adequate face and content validity. The mean overall rating by periodontists was lower than dental students, 72.1 ± 15.8 vs. 83.7 ± 9.7; 95% CI: 3.36–19.85, *p* < 0.001.

Looking at individual questions, periodontists gave significantly lower ratings than students for questions evaluating model realism, the tactile sensation of the incision on the models, as well as flap reflection sensation and the authenticity and overall simulation experience for presurgical simulation training in oral surgery. The differences were statistically significant for questions 2,6 and 8 in both Mann-whiney U and Welch t-test for questions 2,6 and 8. They remained significant only for questions 2 and 6 based on Bonferroni correction (adjusted α = 0.00625, *P* < 0.006) (Table 3).

**Table 3:** Comparison of student and periodontist groups’ VAS ratings across eight questions and overall mean score. Mean and standard deviation (SD) are reported for each question (Q1–Q8). Independent-samples *t*-tests were used to compare group means. (*p < 0.006)

Question	Students Mean (SD)	Periodontists Mean (SD)	Mean Difference (95% CI)	*p*-Value
Q1 – Anatomical appearance	**85.07 (11.20)**	**78.88 (12.31)**	**6.18 (–0.84 to 13.21)**	**0.064**
Q2 – Tactile sensation of incision	**77.98 (12.83)**	**52.11 (26.92)**	**25.87 (12.1 to 36.64)**	< 0.001 *
Q3 – Flap reflection	**75.50 (16.37)**	**62.50 (25.74)**	**13.00 (−0.46 to 26.46)**	**0.058**
Q4 – Alveolar bone appearance	**88.70 (9.62)**	**84.72 (21.26)**	**3.98 (–6.87 to 14.82)**	**0.817**
Q5 – Suturing simulation	**80.06 (14.85)**	**74.06 (16.23)**	**6.00 (–3.19 to 15.20)**	**0.167**
Q6 – Authenticity for training	**84.68 (11.51)**	**68.89 (19.35)**	**15.79 (5.73 to 25.85)**	< 0.001 *
Q7 – Transferability to clinical training	**84.98 (11.34)**	**75.72 (18.1)**	**9.26 (−0.18 to 18.69)**	**0.079**
Q8 – Overall experience	**93.24 (11.59)**	**80.22 (21.99)**	**13.02 (1.7 to 24.34)**	0.004*
Overall Mean Score	**83.75 (9.67)**	**72.14 (15.83)**	**11.61 (3.35 to 19.85)**	< 0.001*

The questions were grouped into two main domains: those evaluating face validity (Questions 1, 4, and 6), which assessed the realism of the models relative to clinical reality as Face validity, and those evaluating content validity (Questions 2,3,5,7,8), which examined the educational relevance and appropriateness of the model for skill trainings.

**Fig. 4:**
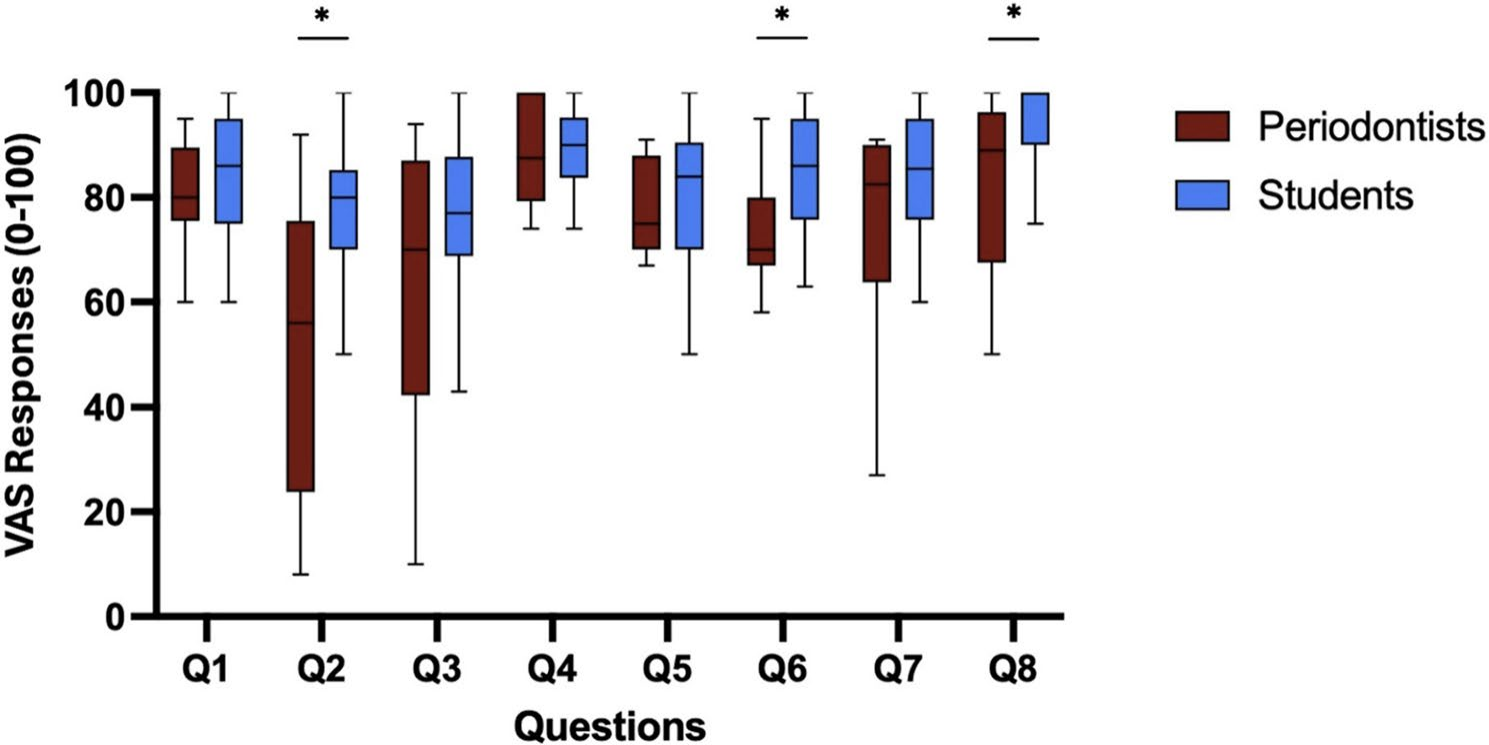
Comparison of visual analogue scale (0–100) responses between student and periodontist groups for individual questions. Q1: Overall anatomical structure and soft tissues., Q2: Tactile sensation of performing an incision, Q3: Tactile sensation of flap reflection, Q4: Appearance of the alveolar bone and its defect morphology, Q5: Simulation of the suturing procedure, Q6: Realistic (Authentic) simulation for presurgical training in oral surgery. Q7: Transferability of the 3D-printed model for simulating presurgical training in oral surgery. Q8: Overall, experience working with such 3D-printed models is an asset to clinical training. Each boxplot illustrates the interquartile range between the 25th and 75th percentiles; the horizontal line within each box represents the median score. The whiskers extend to the most extreme data points within 1.5 times the interquartile range. (*P < 0.006 Bonferroni corrected)

**Fig. 5:**
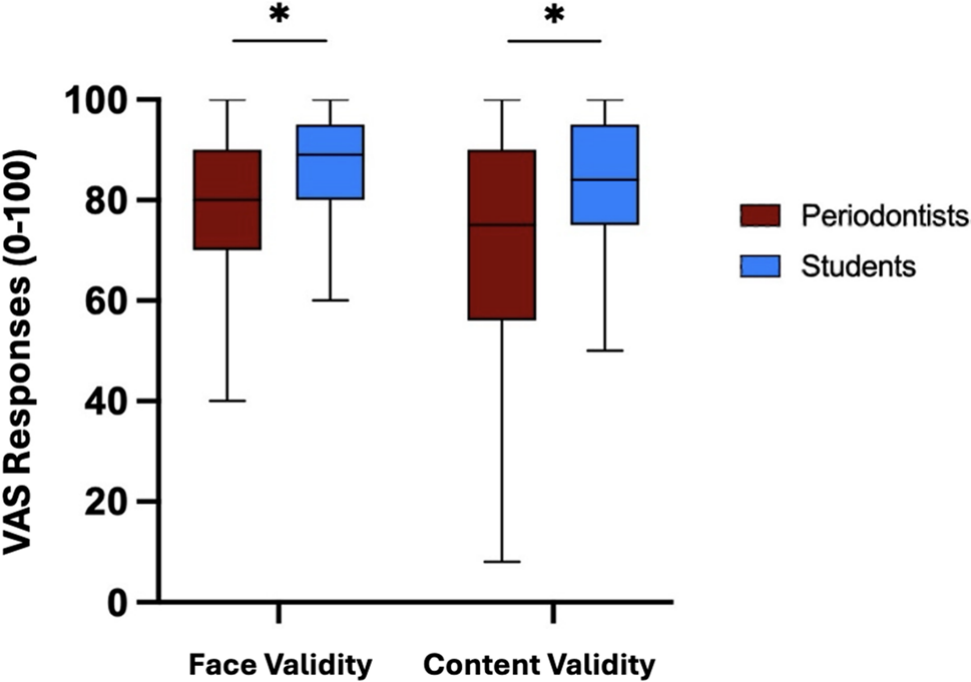
Comparison of Visual Analogue Scale (0–100) responses between student and periodontist groups for questions 1,4,6, assessing the simulation model’s authenticity and questions 2,3,5,7,8, assessing the model transferability. Each boxplot illustrates the interquartile range between the 25th and 75th percentiles; the horizontal line within each box represents the median score. The whiskers extend to the most extreme data points within 1.5 times the interquartile range. (*p < 0.01 Bonferroni corrected threshold)

When analyzing grouped questions’ VAS score, periodontists rated the face validity (of the model significantly lower than students (77.47 ± 19.00 vs. 86.33 ± 10.78), with a mean difference of 8.86%. Transferability score comparison also showed periodontists rates to be significantly lower than students’ (70.13 ± 23.25 vs. 82.75 ± 14.33), with a mean difference of 12.62%. These differences remained statistically significant after Bonferroni correction (*P* < 0.01). Data is reported in boxplots and screened for outliers using standardized z-scores (threshold: *z* ***> ±*** *3.0*), with no data points being beyond the threshold. Each box represents the interquartile range between the 25th and 75th percentiles; the horizontal line within each box indicates the median score. Whiskers extend to the most extreme data points within 1.5 × interquartile range (Figs. 4 and 5).

In the free text comments, participants provided positive feedback on the 3D-printed simulation model, highlighting its value as a training tool. In terms of authenticity or face validity, the model was widely perceived as realistic in appearance and was commended for its accurate representation of bone morphology. However, the soft tissue was considered less representative of clinical conditions, especially in sulcular areas. A few had noted that the soft tissue felt slightly more sticky or rubbery compared to a real gingiva, with flap reflection being easier and incisions more difficult than a real-life counterpart. Additional feedback noted that a dry environment, lacking saliva and blood, was a marked difference when compared to the real-life oral cavity. Overall, in terms of transferability or content validity, the model was regarded as particularly valuable for building learners’ confidence in a risk-free environment. Many participants noted that the skills practiced, especially flap design and suturing, could be effectively translated to clinical scenarios, despite some differences in tactile feedback. To analyze the open question responses, word clouds were generated from participants’ qualitative feedback using the online platform Free Word Cloud Generator (https://www.freewordcloudgenerator.com). Separate visualizations were created for positive and negative descriptors, with word size proportional to their frequency. 

**Fig. 6:**
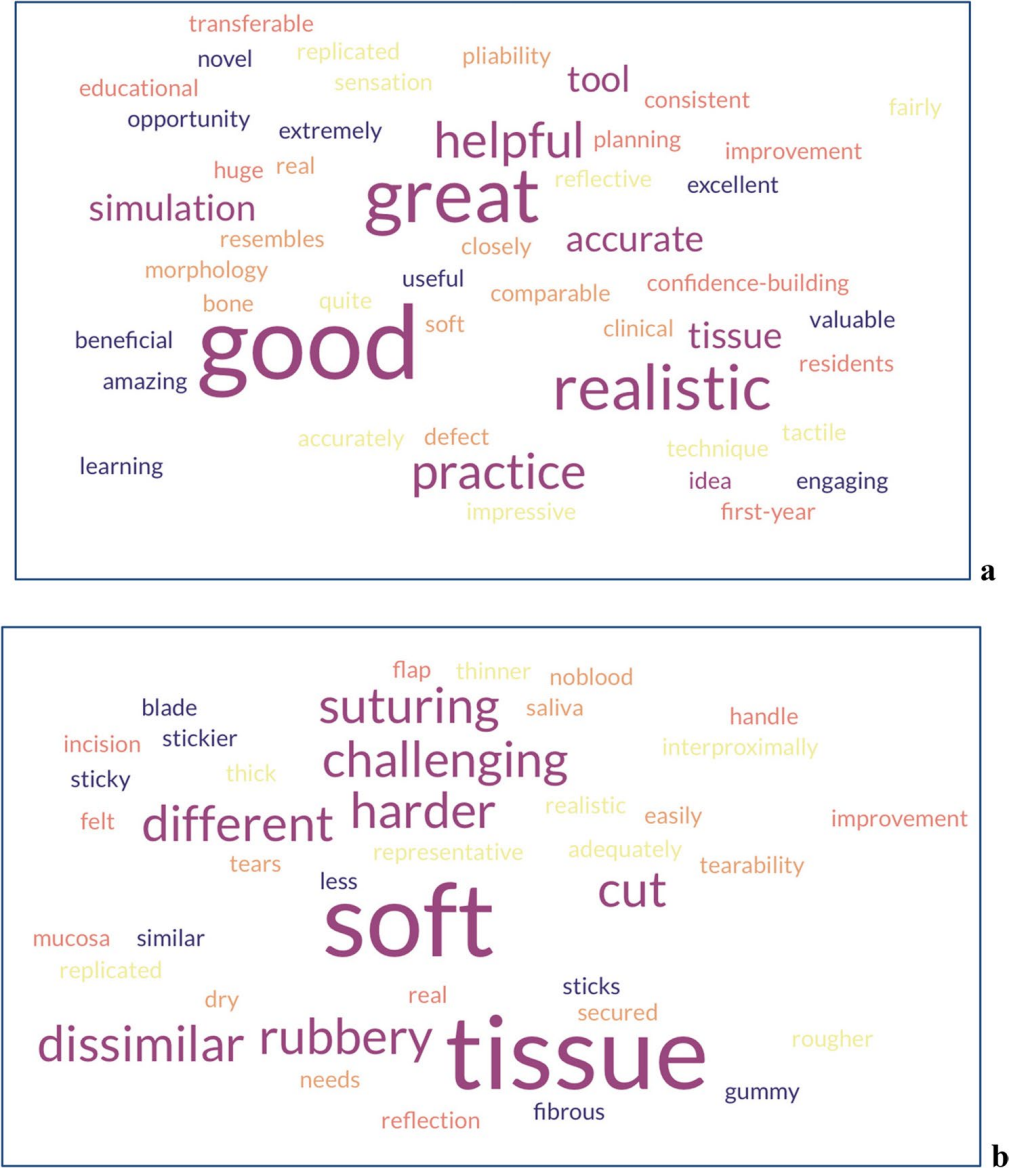
Word clouds of panel **a**: positive adjectives, and panel **b**: negative adjectives used in the free-text feedback on the 3D-printed simulation model

We conducted a retrospective justification of sample adequacy. A post hoc sample size estimation based on the primary outcome (overall VAS rating of model realism/utility: The observed means were 83.7 ± 9.7 for students and 72.1 ± 15.8 for experts, corresponding to an effect size of d = 0.88. For a two-sided independent-samples t-test with α = 0.05 and 80% power, approximately 21 participants per group (42 total) would be required to detect a difference of this magnitude. Our study included 50 students and 18 experts, which is close to the required number and reflects the available population in our program.

## Discussion

Simulation-based training provides learners with the opportunity to develop clinical and surgical skills in a safe, standardized, and repeatable environment. The workflow presented in our study demonstrates a feasible and suitable approach for designing patient-specific, 3D-printed oral surgery models for training clinicians.

The soft tissue material chosen for the models had better stretchability and overall tactile feel compared to the printer’s soft tissue resin and a tested silicone material. The authors believe it can even be considered for various oral surgery procedures, such as guided bone or tissue regeneration and even soft tissue surgery procedures. The soft tissue material could provide suitable stretchability properties for flap advancement and periosteal release incision simulation. This has been an aspect that other researchers have found printable soft tissue materials to lack and have attempted to address with other techniques [11, 12]. However, its broader applicability and educational impact warrant further development and investigation in education settings.

The proposed workflow, integrating intraoral scans with CBCT data, demonstrated a novel approach for fabricating precise patient-specific 3D-printed oral surgery models for both hard and soft tissues. Overall, the amount of accuracy and realism needed for a training model may vary depending on the procedure. Interestingly, in some studies, evaluators have rated the educational utility of 3D-printed models high, even when their anatomical realism received lower scores [16, 18]. This deviation was mainly due to differences in soft-tissue thickness, and these error margins are clinically acceptable for simulation. A reason for this range may be that we used a mould injection for the soft tissues. Furthermore, image processing steps such as surface mesh smoothing of the CBCT may lead to the loss of fine anatomical details. Additionally, limited intraoral scan coverage, particularly in the full depth of the vestibular area, may have impacted accuracy. These factors should be considered for further improving the model’s fidelity [21–26].

The estimated cost per full-arch model with the present workflow is relatively low, especially considering its educational value. While setup and technician costs were not included in this estimation, they can be one-time design costs or shared institutional investments. Developing a reusable model library and customizing prints (e.g., quadrant or sextant size) can further reduce costs. Overall, as technology improves and 3D printer technology costs are reduced, these 3D printed models are becoming more cost-effective tools in various medical fields [6, 10, 15, 27].

Face and content validity are considered critical aspects to evaluate when assessing simulation models used for surgical skill training. The findings aligned with several recent studies that have applied 3D printing to medical imaging data from patients to develop simulation tools. Some previous studies have asked their participants to compare 3D-printed models with cadaveric models. The main finding of these reports has shown 3D-printed models to be rated better in terms of visual realism to human anatomy. Such real patient 3D models provide more detailed information about critical anatomical structures, which cannot be accurately replicated in traditional models; however, they were consistently rated to have lower tactile sensation, especially for soft tissues in several studies [10, 15, 19, 27–29]. Therefore, the search has been ongoing to provide a more realistic replication of human tissue elasticity and, the search has been going on to provide a more realistic realistic replication of human tissue elasticity and adhesion. Jannot et al. found expert ratings to be lower than those of students for the printer’s gingival material. They reported the soft tissue component to be thick and rigid, making it difficult to incise. This was similar to our evaluation of the printable gingival material available for the 3D printer used in the present study [16].

In the present study, the educational value of 3D-printed models was supported by both student and expert evaluations, although experts consistently rated soft-tissue feedback lower. This can reflect their higher expectation. This difference in opinion comes from more experience of surgery on real-life tissues. A few studies have been conducted similar to the present study, asking both expert surgeons and students to evaluate 3D-printed models using questionnaires [10, 16, 29]. These studies have also reported lower ratings by experts than by students, which reflects their higher expectations and aligns with their experience working with real human tissues. This comparative evaluation can provide a valuable perspective on the evaluation of simulation training models. In contrast, dental students, who are still developing their psychomotor and perceptual skills, may perceive the models as sufficiently realistic and educationally valuable. It is advised to consider expert evaluations in study design.

There are a limited number of studies similar to the current research that have considered fabricating 3D-printed models with precise patient-specific soft-tissue replication [16, 28] Jannot et al. presented a workflow to replicate both soft and hard tissues of the patient. They introduced a 3D-printed simulator for the treatment of intraosseous and interradicular lesions, including scaling, incisions, and suturing. They had used a 3D printer (Form 3BL; Formlabs) to print all the soft and hard tissues of the model. The soft tissue used by their group was a printable soft tissue of the Flexible 80 A resin (Formlabs) that was colored pink, and as mentioned, it did not have the suitable criteria to provide an authentic soft tissue sensation.

Recently, Gund et al. developed and validated a 3D-printed model designed to practice hemi-sectioning and bone grafting in a periodontally compromised mandibular molar. They had the model’s face validity and its transferability evaluated only by 14 students using a questionnaire and compared with pig jaw models. The 3D printed model was found to provide high anatomical visualization of the complex bony defects. However, participants noted that soft tissue handling remained more realistic in cadaveric specimens [28]. Unlike our models, they had not used any intraoral scan or printing to replicate the soft tissues of the model. Instead, Pink cotton-lined gloves (Spontex Feeling, MAPA GmbH, Germany) were cut and bonded to the PLA ridge using reversible rubber adhesive (Fixogum, Marabu GmbH) to simulate detachable gingival soft tissue.

Although a similar 3D-printed molding approach was tested by Antunes et al., they did not utilize a patient’s actual intraoral scan data; instead, their mold was created by digitally enlarging mandibular CBCT scan data. In contrast, our proposed workflow enabled precise replication of gingival tissues overlying the bone.

Additional perspectives noted in the present study and the similarly by Antunes et al. was the participants’ feedback of a lack of saliva and blood, which reduces clinical realism. These can be considered as other areas for future innovation in simulation design [17]. Moreover, in the free text comments, lack of saliva and blood was mentioned as a difference from clinical reality. In an innovative approach, Smail et al. have recently developed and evaluated an intraoral model that can replicate bleeding when incision is done. Their model received positive feedback from all participants and was considered a reproducible, ethically sound alternative to animal models [29].

Collectively, the available literature in this field, in conjunction with the current study, supports the notion that 3D-printed models show promise for use in oral surgical skill development. However, replicating precise intraoral soft tissue characteristics remains challenging and various researchers have come up with their methods of replicating soft tissues. This limitation has been recognized by researchers, highlighting an important area that needs further material improvement and innovation [12, 13, 15–17].

### Limitations and future directions

This exploratory study demonstrated the feasibility and educational potential of 3D-printed patient-specific simulators. Although evaluation relied primarily on subjective VAS feedback, these insights provide a solid foundation for future validation. A post hoc power analysis indicated adequate power (80%) to detect the observed group differences in overall model evaluation scores. However, future studies with larger sample sizes or multicentre cohorts that incorporate objective skill assessments can strengthen the evidence towards the implementation in surgical training curricula. Moreover, a longitudinal study design that assesses repeated simulator use could clarify the impact of these tools on long-term skill retention and clinical performance. Future investigation on the model’s application across diverse anatomical and clinical scenarios and the refinement of haptic fidelity, particularly in simulating soft-tissue properties, remains an important goal.

Use of multi-material 3D printing and innovative methods to provide dynamic fluid simulation (e.g., saliva or blood flow) or to integrate in mixed reality environments are exciting opportunities to be considered for enhancing the realism and creating next-generation, high-fidelity surgical training models [30].

## Conclusion

The fabrication process in the current study closely replicates the patient’s hard and soft tissue in the 3D-printed model. The model demonstrated promise as an educational tool for presurgical planning and simulation of oral surgery procedures such as flap elevation and suturing. Feedback from both students and periodontists was generally positive, suggesting its potential value in supporting surgical training and enhancing learning experiences in dental education.

## Data Availability

The datasets used and/or analysed during the current study are available from the corresponding author on reasonable request.

## Abbreviations

3D: Three–Dimensional
VAS: Visual Analogue Scale
CBCT: Cone–Beam Computed Tomography
DICOM: Digital Imaging and Communications in Medicine
STL: Standard Tessellation Language
SPSS: Statistical Package for the Social Sciences
SD: Standard Deviation
CI: Confidence Interval
